# Propidium monoazide pretreatment on a 3D-printed microfluidic device for efficient PCR determination of live versus dead’microbial cells†

**DOI:** 10.1039/c8ew00058a

**Published:** 2018-06-11

**Authors:** Yanzhe Zhu, Xiao Huang, Xing Xie, Janina Bahnemann, Xingyu Lin, Xunyi Wu, Siwen Wang, Michael R. Hoffmann

**Affiliations:** a Linde + Robinson Laboratories, California Institute of Technology, Pasadena, California 91125, USA; b School of Civil and Environmental Engineering, Georgia Institute of Technology, Atlanta, Georgia 30332, USA; c Institute of Technical Chemistry, Leibniz University, Hannover, Germany

## Abstract

Waterborne microbial pathogen detection via nucleic acid analysis on portable microfluidic devices is a growing area of research, development, and application. Traditional polymerase chain reaction (PCR)-based nucleic acid analysis detects total extracted DNA, but cannot differentiate live and dead cells. A propidium monoazide (PMA) pretreatment step before PCR can effectively exclude DNA from nonviable cells, as PMA can selectively diffuse through compromised cell membranes and intercalate with DNA to form DNA–PMA complex upon light exposure. The complex strongly inhibits the amplification of the bound DNA in PCR, and thus, only cells with intact cell membranes are detected. Herein, this study reports the development of a microfluidic device to carry out PMA pretreatment ‘on-chip’. Chip design was guided by computer simu-lations, and prototypes were fabricated using a high-resolution 3D printer. The optimized design utilizes split and recombine mixers for initial PMA-sample mixing and a serpentine flow channel containing her-ringbone structures for dark and light incubation. On-chip PMA pretreatment to differentiate live and dead bacterial cells in buffer and natural pond water samples was successfully demonstrated.

Water impact

We have designed a microfluidic chip for live/dead cell differentiation with propidium monoazide pretreatment. The chip involves computer-aided design of microfluidic structures, and was prototyped by 3D printing. On-chip live/dead cell differentiation was successfully demonstrated for lab and environmental samples. The design can potentially be integrated into a microfluidic microbial monitoring system and advance the accuracy of water risk assessment.

## 1 Introduction

Due to the poor water and sanitation conditions, the out-break of waterborne diseases claims millions of lives per year in many developing countries and countries in conflicts (e.g., the cholera epidemics in Yemen and Haiti).^1,2^ Compared to traditional culture-based methods, polymerase chain reaction (PCR) technology significantly improves the accuracy and sensitivity of pathogen detection and it reduces the analytical time from days to hours.^3^ In recent years, the emergence of microfluidic technologies has enabled the miniaturization of PCR processes onto chip-based devices. Studies have demonstrated automated PCR systems that integrate DNA extraction, thermal cycling, and results reading.^4–6^ These portable systems have shown great potential in waterborne pathogen detection and monitoring, especially in low-resource settings.

Bacterial cells, constituting a major category of waterborne pathogens, can exist in three states characterized by distinct cell behaviors in traditional culture-based methods. The three states are culturable, dead, and a dormancy state called viable but non-culturable (VBNC).^7^ Pathogenic bacterial cells in both culturable and VBNC states pose potential risks to public health, thus should be considered as “live” cells in environmental monitoring and microbial risk analysis. Culture-based methods obviously tend to underestimate the pathogen concentrations due to the presence of VBNC cells under various environmental stresses.^8,9^ However, the differentiation between live and dead cells is even more challenging without cultivation. Although PCR is becoming the new standard in environmental microbial detection, it cannot differentiate live and dead pathogens, since it indiscriminately detects all target DNA fragments in a sample. Studies have shown that a considerable fraction of pathogens in environmental water samples may have lost viability, but their DNA may still be present and detectable by PCR for several weeks.^10^ This would likely result in an overestimation of potential health risks. A few studies showed that dielectrophoresis can separate live and dead cells based on the different induced electrophoretic forces on the cells.^11–13^ The viability of cells may also be assessed by the integrity of their plasma membranes. A combination of two fluorescent dyes SYTO-9 (stains all cells in green) and propidium iodide (only penetrates cells with dam-aged membranes and labels them in red) has been widely used for microscopic and flow-cytometric live versus dead cell determination.^14–16^

Using the membrane exclusion properties of live cells, propidium monoazide (PMA), a DNA intercalating dye, has been coupled with PCR to detect only live cells.^17^ The afore-mentioned dye is able to penetrate the compromised cell membranes of dead cells but not those of the live cells. With light exposure, the azide group on the dye molecule is converted into a reactive nitrene intermediate, which irreversibly forms C–N covalent bonds with adjacent DNA.^18^ Dye-bound DNA loses its ability to bind PCR primers and thus cannot be amplified during PCR cycles. The excess dye mole-cules react with water during light incubation and lose their ability to bind amplified DNAs.^19^ This method intrinsically enables selective detection of live cells including those in VBNC state. It should be noted that the efficacy of PMA pretreatment is not universal among all microorganisms. Some live cells, such as Bacillus subtilis and Staphylococcus epidermidis, have been shown to have a non-negligible PMA permeability, and thus the PMA method may underestimate the number of live cells of such speices.^20,21^ Moreover, not all dead cells exhibit a higher PMA permeability than the live ones. This is particularly true in the case of UV disinfection process, in which the cell death is mainly induced by dam-ages to DNA/RNA instead of to cell membranes. The intact membranes of such dead cells could obstruct the permeation of PMA, leading to an overestimation of live cell concentration. Nevertheless, PMA pretreatment is still applicable and possibly the most rapid method to differentiate live/dead cells when the assay is properly designed.

Traditional PMA pretreatment is performed in-tube by adding PMA into samples, followed by a brief vortex, a given time of dark incubation, and then a light incubation.^22^ Performing PMA pretreatment on a microfluidic chip can eliminate the need of multiple manual pipetting steps with the advantage of accuracy and reproducibility. Moreover, an on-chip PMA pretreatment may also benefit future design of an integrated PMA-PCR microfluidic system. In a micro-fluidic chip with limited channel volume, sufficient incu-bation time requires relatively low flowrates. However, mass transport under these conditions is dominated by diffusion due to small Reynold's and Peclet numbers. To ensure the effective diffusion of PMA into compromised cells, a split and recombine (SAR) mixer can be used to shorten the mixing channel lengths.^23^ However, the multilayer structure of such mixers poses a major challenge for chip fabrication. Conventional fabrication methods such as soft lithography or direct etching are essentially 2-dimensional, which limit the multi-dimensional design. Furthermore, bonding of corresponding channels leads to low turnover rates and poor prototype consistency.^24^

The overall goal of this project is to develop a microfluidic chip to simplify PMA pretreatment in PCR-based live/dead bacterial cell differentiation. COMSOL Multiphysics simulation software was employed to guide the chip design by modeling the fluidic behavior under experimental conditions. High-resolution 3D printing techniques were used to fabricate chips with complicated 3D structures without using traditional clean room facilities. PMA pretreatment to differentiate live and dead bacterial cells in buffer and natural pond water samples was successfully demonstrated using the prototype chip.

## 2 Materials and methods

### 2.1 Flow simulation

The number of split and recombine (SAR) mixer units required for adequate mixing were determined by flow simulation in COMSOL Multiphysics.^25^ The inlet PMA concentration was set as 400 μM, while its concentration at the sample inlet side was set as zero. The fluid properties of bacterial suspension were assumed to be the same as water. The geometry of 15 SAR mixers was assembled in COMSOL and the cross-sectional PMA concentration profiles were simulated at the end of each mixer. COMSOL solves the laminar flow profile of the system and then solves the transport of dilute species. With a PMA to sample flowrate ratio set at 1 : 4, total flowrates of 7.5, 12.5, 25, 50, 100, and 150 μL min^−1^ were tested. At the cross-sections after each mixer unit, the values of |C^PMA^ – 80 μM| were calculated and averaged for all mesh points in the plane, which represents the cross-sectional aver-aged absolute difference between actual PMA concentration (C^PMA^) and target PMA concentration (80 μM). The effective-ness of mixing after certain number of mixers (N) was quantified by percentage of mixing, calculated by eqn (1):

$$\%{\text{Mixing}}_{N}=1-\frac{{\left(|C_{\text{PMA}}-80\mu \text{M}{|}_{n=N}\right)}_{\text{cross-sectional average}}}{{\left(|C_{\text{PMA}}-80\mu \text{M}{|}_{n=0}\right)}_{\text{cross-sectional average}}}$$( 1 )

The design of mixers is based on the simulated percentage of mixing values, which approaches 100% when the fluids are perfectly mixed.

### 2.2 Chip design, fabrication and characterization

**Fig. 1:**
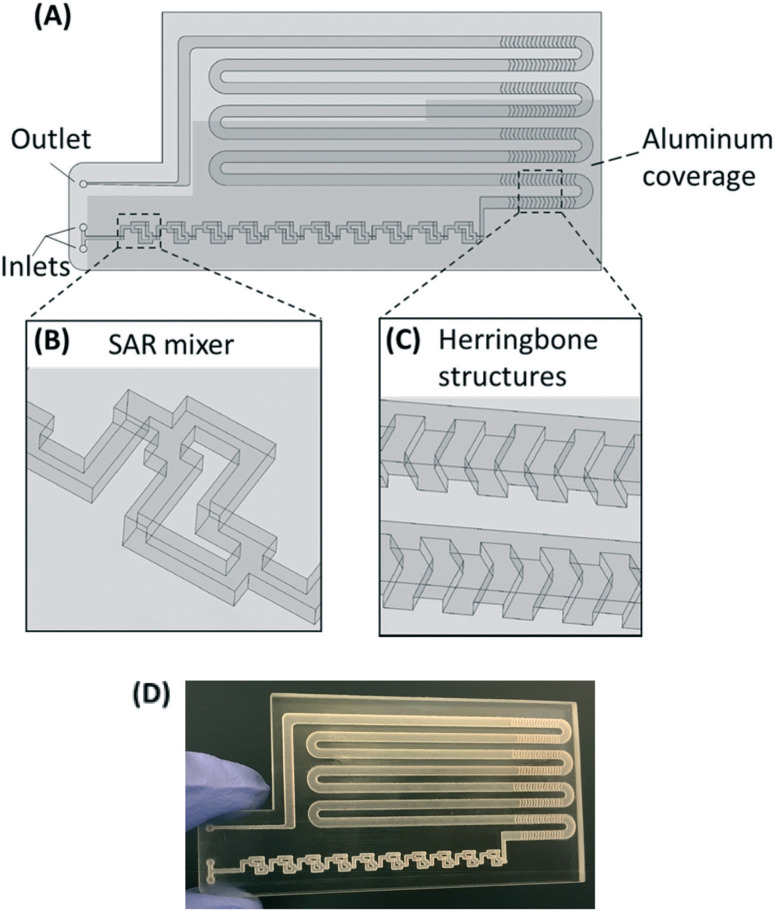
(A–C) Chip design: top view (A) with a zoomed-in 3D view of an SAR mixing unit (B) and the herringbone structures (C). The injected cell sample and PMA solution are continuously mixed in the SAR mixers and then enter the incubation channel with herringbone structures. The darker shade indicates that part of the chip is covered by aluminum foil for dark incubation. (D) Photo of the 3D printed chip prototype.

The PMA pretreatment chip was fabricated using a high-resolution 3D printer (3D systems ProJet™ MJP 2500 Plus, Rock Hill, SC) with clear plastic 3D printing material (Visijet M2 RCL, 3D Systems). After printing was completed, the chip was cleaned in hot mineral oil bath and the channel was flushed with hot mineral oil to remove the supporting wax outside and inside the chip. A schematic diagram and a photograph of chip are shown in Fig. 1A–D. The microfluidic chip contains 10 SAR mixers followed by a serpentine-shaped incubation channel. Herringbone structures were also incorporated into the incubation channel to reduce the residence time difference caused by the parabolic flow profile.^26^

The channel width of the SAR mixer is 500 μm, while that of the incubation section is 2 mm. The total void volume within the chip was calculated to be 138 μL. The thickness of the covering layer above the incubation channel was 0.8 mm. To test the channel integrity and quality, a 500 μm-thick slice was printed with square holes with sides of 500 μm and was viewed under a microscope (Leica M205FA, Buffalo Grove, IL). The light transmittance of the material was assessed by UV-vis spectrometer (Shimadzu UV-2101PC, Kyoto, Japan) over the wavelength range from 200 to 700 nm. A 0.8 mm-thick slide, which has the same thickness as the top cover layer over the channels, was printed. The slide was measured with one side rubbed with mineral oil facing the light source, in order to simulate the chip layer above the fluid during light incubation.

### 2.3 Cell cultures and natural water samples

Escherichia coli (E. coli, ATCC 10798) was employed as model bacteria and cultivated in Luria-Bertani (LB) broth in an incu-bator shaking at 200 rpm for ∼16 h at 37 °C. E. coli cells were harvested and washed 3 times with 1× phosphate-buffered saline (PBS) and used as stock solution. Buffer samples were prepared with 7 × 10^8^ CFU mL^−1^ live E. coli cells spiked in PBS, as estimated by plate counts. To prepare dead cell samples, the stock solution was heat-treated in 90 °C water bath for 10 minutes, and the cell inactivation by this heating protocol was verified by Xie et al.^20^

To investigate the performance of PMA chip in real water samples, environmental water samples were collected from the Turtle Pond in Caltech. The basic water quality parameters are presented in Table S1.†

### 2.4 PMA pretreatment on the prototype chip

The inlets, SAR mixers, and part of the incubation channel were covered with aluminum foil for dark incubation, while the rest of the incubation channel was left uncovered for light exposure (1100 W m^−2^, ABET Sun 2000, Milford, CA). The chip surface facing the light source was rubbed with mineral oil in order to enhance the light transmittance. 400 μM PMA solution (Biotium Inc.) and the water sample were introduced into each inlet via microfluidic connectors (Dolomite M1 4-way linear connector, Royston, UK) with a flowrate ratio of 1 : 4, and the flows were controlled by syringe pumps (Cole-Parmer 74 905-02, Vernon Hills, IL). Depending on the flow rate, usu-ally 1–10 mL cell sample was loaded for each experiment. After injection, the PMA solution and E. coli were mixed in the SAR mixers, followed by dark incubation to allow the diffusion of PMA molecules through dead cell membranes, and then light exposure to induce the reaction between PMA and adjacent DNA. Samples (100 μL per sample) were collected from the outlet in triplicates after the flow reached steady state.

The on-chip PMA pretreatment was first tested with PBS buffer seeded with all dead cells at total flow rates of 7.5, 12.5, 25, 50, 100, and 150 μL min^−1^, corresponding to light exposure time (1/2 total residence time) of 9.17, 5.50, 2.75, 1.38, 0.69, and 0.46 minutes. Control experiments without PMA treatment were performed, in which the PMA solution was substituted by Milli-Q water, with all other conditions kept the same. For in-tube PMA pretreatment, 20 μL 400 mM PMA solution was mixed with 80 μL cell solution. Then the samples were incubated in dark for designated time before light incubation. The dark/light incubation conditions tested were the same as the on-chip experiments.

At the optimal flowrate for dead cell discrimination, live and dead E. coli cell mixtures (100% live, 10% live, and 100% dead) in PBS were tested on-chip and in-tube. For natural pond water, a mixed sample with 90% heat-treated pond water and 10% non-treated pond water is prepared. The mixed pond water samples were tested under the optimal flow condition on-chip with PMA, in-tube with PMA, and on-chip without PMA.

### 2.5 PCR assays

Sample DNAs were extracted with a commercial DNA extraction kit following the instructions (PureLink^®^ Genomic DNA Mini Kit, Thermo Fisher Scientific) and quantified by real-time PCR following the similar protocol as Xie et al. (Eppendorf 6300 Realplex 2, Hamburg, Germany).^20^ Each 20 μL reaction mixture consists of 10 μL PerfeCTa® qPCR ToughMix® (Quanta BioSciences Inc.), 0.25 μM forward primer, 0.25 μM reverse primer, 0.25 μM TaqMan probe, 2 μL DNA sample, and nuclease free water. The real-time PCR analysis was targeting the universal bacterial 16S rRNA gene. These-quences of the primers and the probe used are listed in ESI.†

The software (Eppendorf Inc.) accompanied the real-time PCR instrument was used to evaluate threshold cycle (C^t^ values). The sample DNA concentrations are reflected by threshold cycle (C^t^ values), where larger C^t^ value indicates lower DNA concentration. The effectiveness of on-chip PMA pretreatment, as well as in-tube pretreatment, was showed by ΔC^t^, which was calculated by subtracting C^t^ values of the cell sample before PMA treatment (C^in^ ) from those of sample after PMA treatment (C^t^_out_) (corrected by the dilution ratio of mixing units required is demonstrated quantitatively in 5/4), as represented by eqn (2):

$$△C_{\text{t}}=(\left(C_{{\text{t}}_{\text{out}}}-{\text{log}}_{2}(\left(\frac{5}{4},),)\right)\right)-C_{{\text{t}}_{\text{in}}}$$( 2 )

## 3 Results and discussion

### 3.1 Simulation of PMA-sample mixing by SAR mixers

**Fig. 2:**
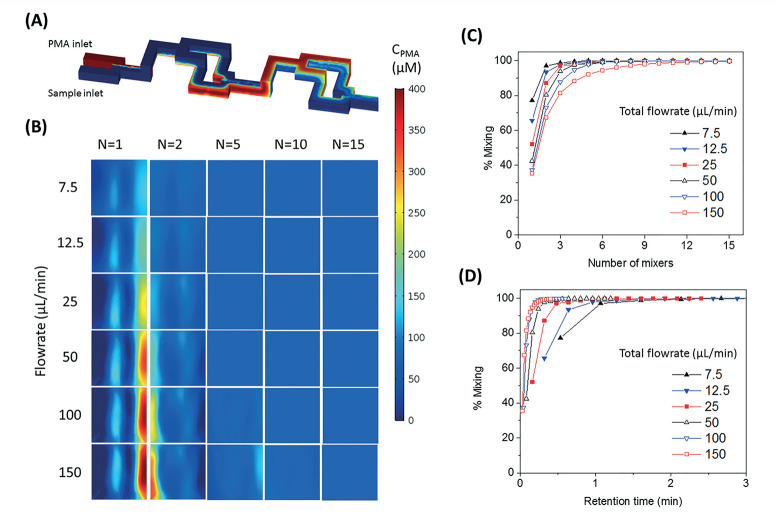
(A) Demonstration of SAR mixing mechanism: simulated cross-sectional concentration of inlets and the first two mixers at a flowrate of 150 μL min^−1^. (B) Simulated PMA concentration profiles at cross sections after 1, 2, 5, 10, and 15 SAR mixers at flowrates ranging from 7.5 to 150 μL min^−1^. (C and D) Simulated percentage of mixing plotted over number of mixing units passe d (C) and total retention time in the mixers (D).

The SAR mixers aid diffusion by “folding” the joined flows, as demonstrated by Fig. 2A.^27^ The mixers utilize the no-slip bound-ary characteristic of laminar flow to re-orient the split flows by a 90° turn, which forces the concentrated side meet with the di-luted side when the flows recombine. The required diffusion length to achieve a well-mixed state is then reduced to facilitate the diffusive transport of PMA molecules. The cross-sectional PMA concentration profiles after 1, 2, 5, 10, and 15 SAR mixers are shown in Fig. 2B. The mixing effectiveness is visually rep-resented by the uniformity of blue color. At the same geometric position (e.g., Fig. 2B, N = 2), fluids under a slower flowrate displayed better-mixed concentration profiles, but the time required to achieve good mixing was much longer.

The tradeoff between time effectiveness and number of mixing units required is demonstrated quantitatively in Fig. 2C and D. Percentages of mixing extent were plotted against the number of mixing units passed and total retention time in the mixing section, respectively. For the same amount of time, higher flowrates result in more efficient mixing, due to more mixing units passed with enhanced advection. However, the optimal flowrate should also take residence time into consideration, since optimal pretreatment requires proper dark and light incubation time for PMA–DNA interaction. Ideally, the design of the mixers needs to provide complete mixing under a range of flow conditions. For the flowrate range of 7.5 to 150 μL min^−1^, which corresponds to a reasonable incubation time range of 9.17 min to 0.46 min,^20^least 10 mixers at were required to reach a plateau with a 98.5% mixing level. Therefore, mixers 10 were employed in our prototype for additional experiments.

### 3.2 Chip fabrication

**Fig. 3:**
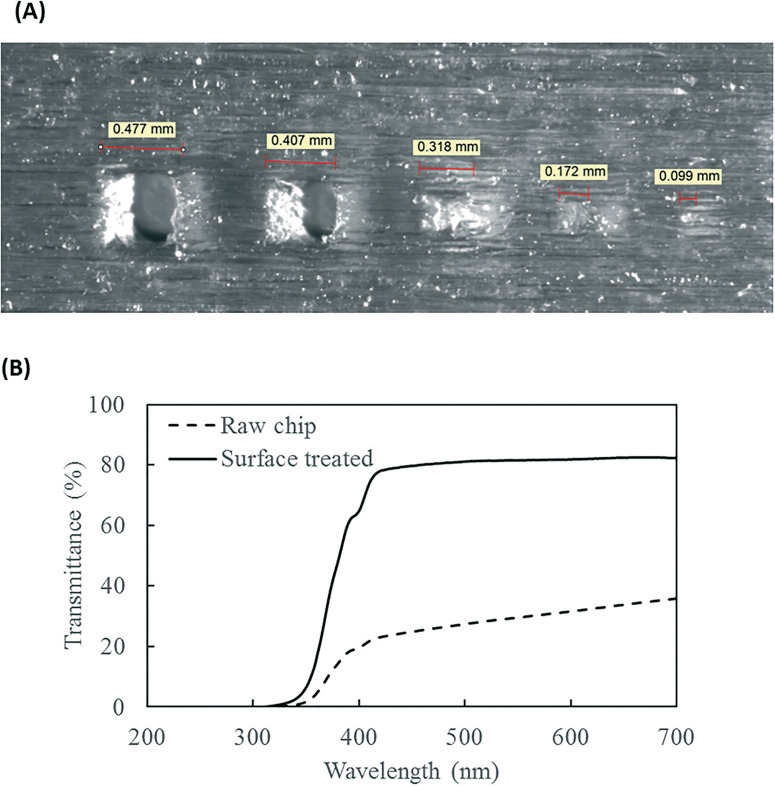
(A) 3D printed channels at designed sizes of (from left to right) 500 μm, 400 μm, 300 μm, 200 μm, and 100 μm. (B) Light transmittance of the 3D printed chip, with and without rubbing mineral oil onto surface, over 200 to 700 nm wavelength range.

The resolution of the 3D printer is 800 × 900 × 790 DPI, which converts to approximate dimensions of 32 × 28 × 32 μm.^28^ The smallest feature of the chip is the SAR channels with 500 μm in size. As shown in Fig.3A the 3D printer can create channels with sides as small as 400 μm and surface roughness less than 30 μm. This demonstrates the feasibility of using the 3D printer to realize the current design of the PMA pretreatment chip as well as some other chips with more complicated microscale structures.

The light transmittance of the 3D printing material was characterized to test its effect on radiation available to the fluids. The freshly printed chip is visually opaque before any surface treatment, due to light scattering by the rough surface produced from 3D printing. Demonstrated in Fig. 3B, the test chip without surface treatment allows less than 30% light transmitted at the optimum wavelength of 470 nm. In contrast, the surface-treated chip has enhanced light transmittance of approximately 80% at the same wavelength. With the surface roughness mostly overcome by applying an oil layer, the loss of transmitted radiation was likely due to absorption by the 3D printing material.

It should be noted that the device is not autoclavable, as the 3D printing material is subject to heat distortion at elevated temperature. However, the material is resistant to common solvents like ethanol and isopropanol, which can be used for cleaning and sterilization purposes.^28^ The material cost of a single microfluidic chip is around $5. Compared to traditional microfluidic chip fabrication methods, 3D printing provides a fast and cost-effective way for prototyping.

### 3.3 Performance of on-chip PMA pretreatment

**Fig. 4:**
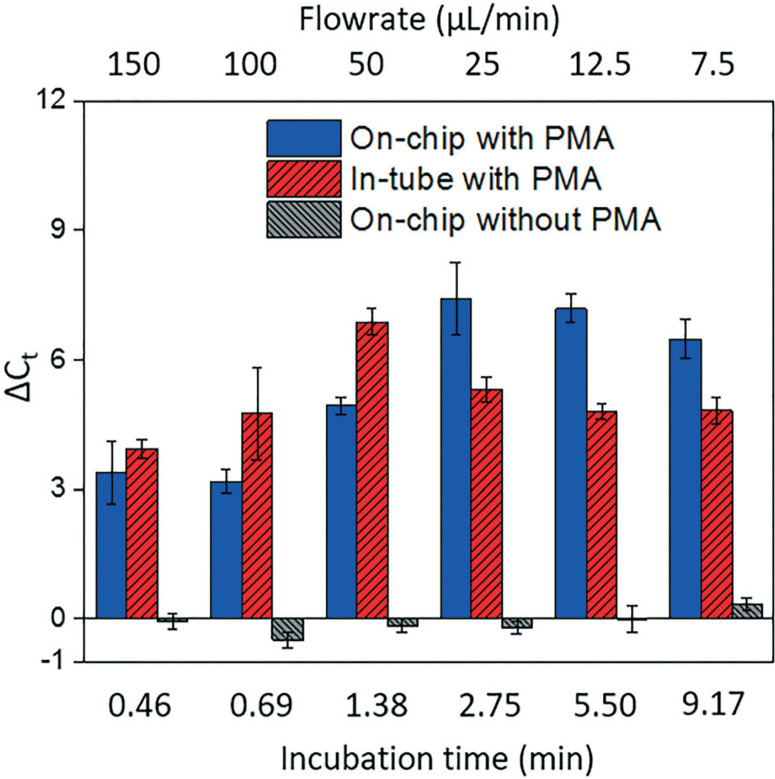
The effectiveness of dead cell discrimination with PMA treatment. The experimental results were quantified by real-time PCR and expressed as the differences in the cycle number (ΔC_t_) for samples before and after PMA pretreatment. The performance was tested un-der various flowrates, which corresponds to different light incubation time. The error bars represent the standard error (SE) of ΔC_t_.

Fig. 4 shows dead cell discrimination was achieved with the designed chip. ΔC^t^ values for various dark/light incubation times are reported along with those acquired for in-tube and on-chip no-PMA control experiments. The higher ΔC^t^ value indicates larger PCR signal reduction (dead cells' DNA was successfully blocked). For in-tube experiments, the peak ΔC^t^ observed was at an incubation time of 1.38 minutes. A simi-lar maximum ΔC^t^ value was also observed by Xie et al., at a light incubation time of 2 min, while the ΔC^t^ declines with extended light exposure likely due to the degradation of the PMA–DNA complexes.^20^ A similar trend was observed in on-chip experiments, with the optimal performance (ΔC^t^ = 7.41 ± 0.85) at the incubation time of 2.75 minutes (corresponding to the flowrate of 25 μL min^−1^). The result was not significantly different (t-test, p > 0.5) from the optimal performance in the in-tube pretreatment (ΔC^t^ = 6.88 ± 0.31 at 1.38 min). The slight delay in attainment of an optimal performance is likely related to the light exposure efficiency.

The tubes employed in in-tube experiments were made of highly transparent polypropylene,^29^ while the roughness and light absorbance of the 3D printing material compromised a small fraction of radiation, so that longer incubation time was required to obtain the optimal on-chip PMA pretreatment. In on-chip no-PMA control experiments, the ΔC^t^ values were closed to 0. This result implies that very few dead cells were lost during on-chip treatment due to trap-ping and sedimentation. Therefore, the increase observed ΔC^t^ values in on-chip PMA treatment can be attributed to PMA pretreatment.

**Fig. 5:**
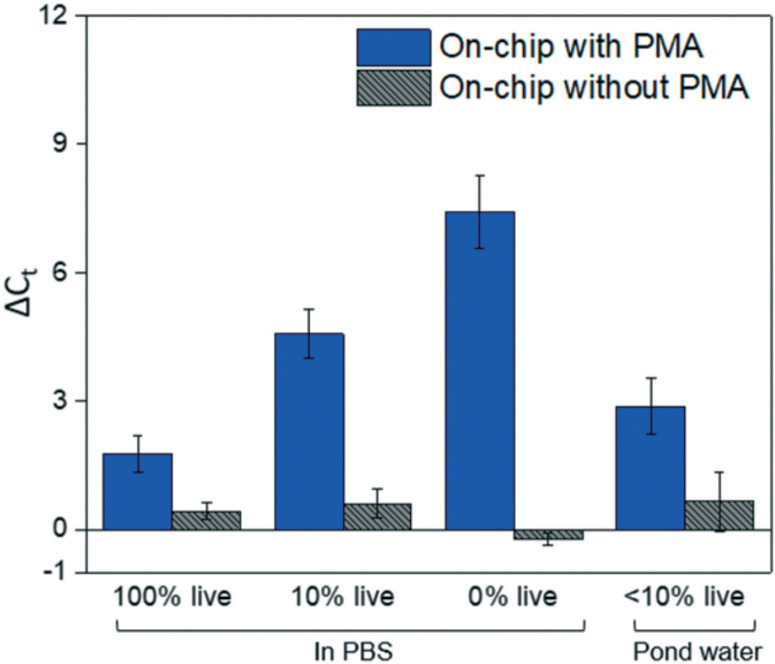
The on-chip PMA pretreatment performance, as well as no-PMA control, for live cells, dead cells, 10% live cell samples, and 10% live pond water samples under the optimal incubation time as previously determined. The results for dead cells under the same condition are plotted as a comparison. The error bars represent the SE of ΔC_t_.

At the optimal flowrate (25 μL min^−1^), the on-chip differentiation of live and dead bacterial cells in the spiked buffer samples and natural pond water samples are demonstrated in Fig. 5. For 100% live cells, on-chip PMA treatment resulted in a ΔC^t^ of 1.77 ± 0.43. This value is significantly smaller (t-test, p < 0.01) in comparison to the ΔC^t^ observed in dead-cell-only samples (ΔC^t^ = 7.41 ± 0.85). This indicates that the on-chip pretreatment significantly discriminated dead cells while causing much smaller change in the reading of live cells. For a mixed cell sample with 10% live cells and 90% dead cells, deactivation of 90% initial DNA templates would result in a ΔC^t^ of 3.3 theoretically. The on-chip pretreatment leads to a ΔC^t^ of 4.57 ± 0.58, with the elevated ΔC^t^ likely attributed to the some of the live cells were also blocked by PMA. For the pond water, the total indigenous microbial population was analyzed as the PCR primers targeted at the universal bacterial 16S rDNA. The preliminary result showed that ΔC^t^ was in the range of 0.58 to 1.28, which may not be reliable as it runs close to the detection limit of the assay. Real-time PCR analysis can differentiate as little as two-fold target gene changes. In PMA pretreatment, this indicates that dead cells in the sample have to be more than 50% (total DNA : live cell DNA >2 : 1), corresponding to a ΔC^t^ increase larger than 1 after PMA pretreatment. Therefore, a mixture of heated and unheated (v/v = 9 : 1) pond water sample was prepared to better demonstrate the on-chip PMA treatment. PMA pretreatment on-chip yields ΔC^t^ of 2.88 ± 0.65, indicating dead cells constitute 89.6–91.3% of the total microbial population. This result generally agrees with the preliminary estimation that 90–96% cells were dead in the sample. The slight deviation from prediction might be due to insufficient PMA pretreatment as the pretreatment conditions were not optimized for the variety of bacterial species present. Hence, species-specific and matrix-specific optimization would benefit environmental applications.

## 4 Conclusions

The on-chip PMA pretreatment achieves effective dead cell discrimination against live cells in the tested buffer and environmental water samples. Despite discussed limitations, on-chip PMA pretreatment has the advantage of less manual labor required and the potential to be incorporated into an integrated microfluidic system for high throughput, accurate, sensitive, and efficient pathogen detection. The research presented here demonstrates the capability of high resolution 3D printing as a microfluidic prototyping method for new chip design and fabrication. The approach has the advantage of easy design and operation, high fidelity, and quick turn-over rates.^24^ However, wider microfluidic application of 3D printing would call for development of new materials, better knowledge of material properties, and standardization of surface treatment techniques to cater to various demands^24,30^ The combination of COMSOL and 3D printing technologies reduces the complexity of microfluidic chip design and prototyping, and therefore lowers the barrier of microfluidics for environmental applications.
