# Supplementary material for: Propidium monoazide pretreatment on a 3D-printed microfluidic device for efficient PCR determination of live versus dead’microbial cells
Source: Environ Sci (Camb). 2018 Jun 11;4(7):956–64. doi: 10.1039/c8ew00058a (PMC7705123; doi:10.1039/c8ew00058a)
Supplement: Supplementary file 1 [file ESWRT-04-956-s001.pdf]

Electronic Supplementary Material (ESI) for Environmental Science: Water Research & Technology.
This journal is © The Royal Society of Chemistry 2018

Supporting Information

Propidium Monoazide Pretreatment on a 3D-printed Microfluidic Device for Efficient PCR Determination of 'Live versus Dead' Microbial Cells

Yanzhe Zhu[1], Xiao Huang[1], Xing Xie[1,2], Janina Bahnemann[1,3], Xingyu Lin[1], Xunyi Wu[1], Siwen Wang[1], and Michael R. Hoffmann[1]*

1. Linde+Robinson Laboratories, California Institute of Technology, Pasadena, California 9112, E-mail: mrh@caltech.edu; Tel: +1-626-395-4391
2. School of Civil and Environmental Engineering, Georgia Institute of Technology, Atlanta, Georgia 30332, E-mail: xing.xie@ce.gatech.edu; Tel: +1-404-894-9723
3. Institute of Technical Chemistry, Leibniz University, Hannover, Germany

PCR conditions, primers and probe

The PCR thermocycling involves 3 minutes of initialization at 95 °C, and 42 cycles of denaturation 95 °c for 15 seconds followed by annealing/extension at 55 °C for 30 seconds. The primers and probe are targeting at the universal 16s rRNA gene. The sequences are listed below.[1]

	Sequence
Forward primer	5’CGGTGAATACGTTCYCGG3’ where Y is either C or T
Reverse primer	5’GGWTACCTTGTTACGACTT3’, where W is either A or T
TaqMan probe	FAM-5’CTTGTACACACCGCCCGTC3’

Supplementary Table

Table S1 Water quality parameters of the pond water tested

pH	7.75	
Electrical Conductivity	925.9	µS/cm
UV254	0.003	
COD	74.7	mg/L

Reference

1 M. T. Suzuki, L. T. Taylor and E. F. DeLong, *Appl. Environ. Microbiol.*, 2000, **66**, 4605–4614.
